# Live Coverage of Intelligent Systems for Molecular Biology/European Conference on Computational Biology (ISMB/ECCB) 2009

**DOI:** 10.1371/journal.pcbi.1000640

**Published:** 2010-01-29

**Authors:** Allyson L. Lister, Ruchira S. Datta, Oliver Hofmann, Roland Krause, Michael Kuhn, Bettina Roth, Reinhard Schneider

**Affiliations:** 1 Centre for Integrated Systems Biology of Ageing and Nutrition, Institute for Ageing and Health, Newcastle University, Campus for Ageing and Vitality, Newcastle upon Tyne, United Kingdom; 2 School of Computing Science, Newcastle University, Newcastle upon Tyne, United Kingdom; 3 QB3 Institute, University of California, Berkeley, Berkeley, California, United States of America; 4 Harvard School of Public Health, Department of Biostatistics, Boston, Massachusetts, United States of America; 5 Department of Computer Science, Free University Berlin and Department of Computational Molecular Biology, Max Planck Institute for Molecular Genetics, Berlin, Germany; 6 Biotec, Technische Universität Dresden, Dresden, Germany; 7 International Society for Computational Biology, La Jolla, California, United States of America; 8 Computational and Structural Biology Unit, European Molecular Biology Laboratory (EMBL), Heidelberg, Germany; Princeton University, United States of America

## Introduction

The International Conference on Intelligent Systems for Molecular Biology (ISMB) 2008 conference in Toronto was probably the first life science conference that saw a meeting report based on microblogging activity. This activity started several days before the conference through discussions on the FriendFeed (http://www.friendfeed.com) platform and the creation of an unofficial room dedicated to the conference. The use of this room led both to a physical meetup and a meeting report [1]. The main focus of the bloggers was their shared interest in science communication. Unsubstantiated opinions and off-topic comments were therefore very limited and the general feeling was that it was a positive experience which brought benefits for the broader conference audience as well as for scientists following the scientific program remotely.

As a result, the International Society for Computational Biology (ISCB), organizers of the Intelligent Systems for Molecular Biology/European Conference on Computational Biology (ISMB/ECCB) 2009 conference decided to actively support future blogging efforts. The live blogging efforts described here can be seen as a model for future conferences, with the organizers providing a tight link between the FriendFeed ISMB/ECCB 2009 room (https://friendfeed.com/ismbeccb2009) and the conference Web site in the ISCB Web portal (http://www.iscb.org/ismbeccb2009/). Talk-specific feeds were created each morning, shortly before the start of the first presentation. The feeds are accessible on the conference pages in the appropriate program sections, with the most recent comments visible on the main ISCB portal entry page. They can also be found on FriendFeed by searching for the author name, the title of the talk or the talk identifier as given in the program. The live blogging event was advertised in various ways: mailings to all attendees and ISCB members, announcements in the printed ISCB newsletter (http://www.iscb.org/images/stories/newsletter/newsletter12-1/index.htm), links from the portal and conference Web sites, advance notification of journalists, and advertisements in The Life Scientists room on FriendFeed (http://friendfeed.com/the-life-scientists). Other platforms such as Twitter (http://www.twitter.com) and personal blogs were used at the conference. Because it is difficult to retrieve Twitter statistics and this platform was not used extensively during the ISMB/ECCB 2009, we focus on the reporting activities in the FriendFeed room.

**Figure 1:**
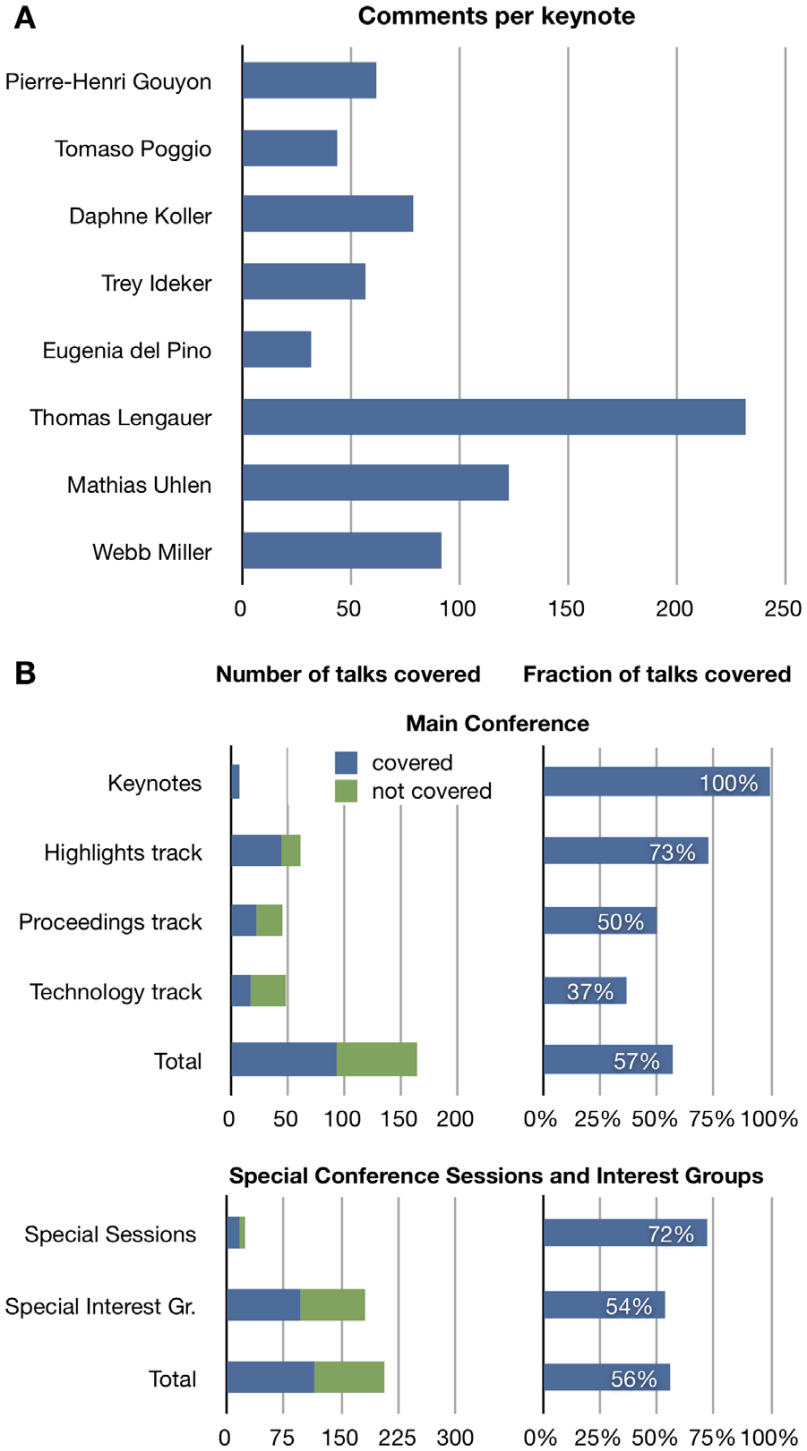
Summary of commented talks at ISMB/ECCB 2009. (A) Keynotes were the most commented talks at ISMB/ECCB 2009. Here, keynotes are listed in chronological order, showing that the number of comments per keynote was higher at the end of the conference than at the beginning. Nonetheless, the absolute number of comments does not necessarily reflect the quality of the coverage. (B) Besides the keynotes, in the main session the highlights and proceedings track received the most attention. However, the total number of covered talks in the SIGs was higher than in any other session. For simplicity, talks from the special sessions and SIGs are summarized across all special sessions and all SIGs, as the commenting method for those sessions was not as uniform as for the main sessions. More detailed statistics are available at the Web sites in Box 1.

More than half of the presentations in the main session (56%, or 93 out of 165) had FriendFeed comments in the ISMB/ECCB 2009 room (see Figure 1), with peak blogging activity during the keynotes. Thomas Lengauer's keynote received the most attention, with more than 230 comments, equating to a comment every 15 seconds. The mean number of comments over all blogged talks in the main session was 22. There were 40 active bloggers, amounting to roughly 3% of conference attendance. There were more than 140 subscriptions to the FriendFeed room, comprising both remote and local attendees.

**Box 1. Session Statistics**

Main session: http://www.iscb.org/cms_addon/friendfeeds/ff_stats.php

Special sessions; http://www.iscb.org/cms_addon/friendfeeds/ff_ssstats.php

SIGs: http://www.iscb.org/cms_addon/friendfeeds/ff_sigsstats.php

Special sessions and main tracks combined: http://www.iscb.org/cms_addon/friendfeeds/ff_statsincss.php

All three session types: http://www.iscb.org/cms_addon/friendfeeds/ff_statsincsssigs.php

Live blogging became so popular at ISMB/ECCB 2009 that, by the end of the first day of the main conference, the security procedures at FriendFeed automatically blocked the IP address of the conference network because of abnormally high activity (http://ff.im/4zHLB). Within about an hour, FriendFeed responded to feedback from conference attendees and reversed the block. To many local and remote attendees, this signalled the beginning of live blogging as a mainstream conference tool (http://blog.openwetware.org/scienceintheopen/2009/06/29/conferences-as-spam-liveblogging-science-hits-the-mainstream/).

## Keynotes

The keynote talks ranged from abstract to applied topics, and from classical biology to cutting-edge computational biology. Pierre-Henri Gouyon, of the Muséum national d'Histoire naturelle, provided the first keynote of the conference, and spoke on the abstract topic of information in biology (*Information and Biology*, http://ff.im/4yWcl) and the difficulty of defining information in a biological context. For Gouyon, biologists are working with fuzzy and poorly defined definitions of information. He describes Richard Lewontin's “triple helix” of genes, environment, and epigenetics as joint providers of information, and warns against simplifying or diminishing the importance of epigenetic information. Gouyon believes that it will take efforts by biologists, computer scientists, and physicists working together to define information in a way suitable for biology.

Tomaso Poggio from the Massachusetts Institute of Technology gave another keynote presentation, *Computational Neuroscience: Models of the Visual System* (http://ff.im/4yVNH). Vision is more than simple categorization: it is also image understanding, inference, and parsing. Poggio has built quantitative models of the ventral stream for object recognition, modelling only the feed-forward connections, and is extending the models to videos and sequences of images. Poggio has tested his models by comparing mouse behaviour, which in turn can be used as a model system for psychiatric diseases. When studying short-duration mouse behaviours, Poggio's system was almost as good as human observers: while classifications by different humans agreed 72% of the time, there was 71% agreement between humans and the system. Further, Poggio's model can be used to infer the mouse strain from behaviours with about 50% accuracy in 10 minutes of video.

Discussing her work on frog developmental biology and how it could be aided by additional computational techniques, Eugenia del Pino from the Pontificia Universidad Católica del Ecuador, spoke about results from frog studies (*The comparative analysis reveals independence of developmental processes during early development in frog*, http://ff.im/4EvDU). del Pino's research focuses on Ecuadorian marsupial frogs (*Gastrotheca*), and how their development compares to other well-studied frog families. Interestingly, the reproductive and egg physiology (i.e., a comparatively larger size of egg), and longer time to the embryonic gastrulation stage of the marsupial frogs bear some resemblance to that of mammals. However, there is a definite lack of molecular data for exotic frogs, and such data could greatly help comparative approaches to frog developmental biology.

Daphne Koller and Trey Ideker described recent advances in networks; while Koller focused on her work with regulatory networks (*Individual Genetic Variation: From Networks to Mechanisms*, http://ff.im/4BFeQ), Ideker discussed the possibilities of using networks for disease diagnosis. Koller, from Stanford University, discussed how, by creating a framework for modelling gene regulation, diverse regulatory mechanisms can be uncovered and the effect of gene regulation on phenotype can be understood. Koller exploits modularity in regulatory networks, which provides increased statistical power, to predict expression of an entire module as well as uncover coupled regulator programs. For example, the transformation of follicular lymphoma to diffuse large B cell lymphoma occurs in 40%–60% of patients, and though gene-based analysis was inconclusive, a regulatory network model performed well, and may have therapeutic implications using connectivity map analysis.

The ISCB Overton Prize Lecture for 2009 was given by Trey Ideker of University of California, San Diego. Ideker described systems biology as a research area for jacks-of-all-trades, rather than masters of one (*New Challenges and Opportunities in Network Biology*, http://ff.im/4BERS). His main focus was on the emerging field of network-based disease diagnosis. Ideker is moving from network assembly of genome-scale data to network-based study of disease. He described how disease networks can aid diagnosis and provide a functional separation of disease gene families, using as an example his work with protein networks to diagnose breast cancer metastasis. Describing the difficulty of extracting subnetworks or modules from the global “hairball” networks, Ideker even suggested that the hairball might be an essential feature of such networks.

Both Thomas Lengauer and Mathias Uhlen discussed their research on applying computational biology to concrete endeavours. Lengauer, from the Max-Planck Institute for Informatics and a founding member of both the ISCB and the ECCB, provided an overview of his ongoing work in *Chasing the AIDS Virus* (http://ff.im/4EvfT). HIV has a dynamic rate of evolution, and with a turnover of more than 10 million virus particles per day per patient, a drug may be efficient against a wild type strain, but not against the mutants present in the infected population. Lengauer models viral evolution to the resistance using a tree structure, where every branching represents several alternatives for viral evolution. Using this technique, Lengauer has discovered that mutations can confer resistance to one drug but increase sensitivity to others. This result could not have been found with mutation tables. Lengauer described how therapy optimization via viral evolution modelling with their THEO prediction engine supports personalized medicine in HIV/AIDS treatment, and helps the doctor make the difficult decision as to which combinations of drugs to give to AIDS patients. THEO (THErapy Optimizer) has a therapy classification error rate of less than 15%, as opposed to 24% for standard methods.

Mathias Uhlen, of the Royal Institute of Technology in Sweden and inventor of pyrosequencing, believes the 21^st^ century is the century of medicine, and that systems biology will play a strong role over the next 10 years (*A global view on protein expression based on the Human Protein Atlas*, http://ff.im/4Hk76). As proteins are primary drug targets and there is a pressing need for antibodies as protein probes, Uhlen developed the Human Antibody Initiative (HAI), Antibodypedia (http://www.antibodypedia.org), and the Human Proteome Resource (HPR, http://www.proteinatlas.org/). The HAI analyzed the success rate of more than 500 commercially available antibodies from 51 commercial companies, and discovered a wide variety of successes and failures, which can be accessed through Antibodypedia. The HPR is a multidisciplinary collaboration that systematically generates antibodies, producing about 200 clones and 2 Terabytes of data per week.

Webb Miller, who received the ISCB Senior Scientist Accomplishment Award for 2009, gave another keynote (*Bioinformatics Methods to Study Species Extinctions*, http://ff.im/4HjYa). In accordance with the theme of his ISCB award, he began by discussing his 10 steps to success in bioinformatics (http://www.iscb.org/iscb-publications/index.php?option=com_content&view=article&id=324). Specifically, his research focuses on how genomic properties are related to the extinction, or risk of extinction, of a species. Miller has performed sequence analysis for a number of rare or extinct species including the Tasmanian tiger, the woolly mammoth, and the Tasmanian devil. For extinct species, the mitochondrial genome is up to 1,000 times more abundant than genomic DNA and is therefore easier to sequence. Even with the limitations in working with ancient DNA, information can be discovered on phylogenetics, population genetics, the evolution of function, and evolutionary rates. For example, Miller discovered that the Tasmanian Tiger diverged from the Tasmanian Devil about 40 million years ago, and that an epidemic was the probable cause of their extinction in 1936.

## Overall Coverage

Based on the statistics from the FriendFeed room, keynotes were the most commented talks, likely due to the larger number of attendees present (http://www.iscb.org/cms_addon/friendfeeds/ff_stats.php). However, live bloggers attended all tracks, satellite meetings and special interest groups (SIGs), with only a single special session missed. Five out of the nine SIGs and four out of the six special sessions had greater than 50% talk coverage. The technology track, which dealt with advances in implementations and software, was the least well covered. Even so, three of the five most-liked presentations overall were from this track. Eamonn Maguire's discussion of computational biology in the cloud was the third most-liked talk overall (http://ff.im/4Evkq). Janet Thornton's overview of the ELIXIR infrastructure for biological information in Europe (http://ff.im/4yWaK) and Frank Tanoh's presentation on the Web service registry BioCatalogue (http://ff.im/4Evnh) placed fourth and fifth, respectively.

Tobias Marschall's discussion, *Efficient Exact Motif Discovery*, was the most-commented presentation in the proceedings track (http://ff.im/4Evno). Marschall described how measuring and locating over-representation are the main issues in unsupervised automated motif discovery when no previous knowledge is assumed. Here, calculation of probabilities of motifs given query text, an IUPAC motif and a random text model can be difficult due to the large number of available motifs. In the highlights track, Eric Alm of MIT spoke about ecological and genetic diversity (*Modeling Ecological and Genetic Diversity in Bacteria*, http://ff.im/4yWcn). Alm and his group, working with Martin Polz at MIT, collected four buckets of seawater off the coast of Massachusetts, two in the fall and two in the spring. They noticed that the organisms clustered seasonally and discovered that the free-living (small-particle) organisms clustered separately from those attached to zooplankton. Many of the groups consisted of only *Vibrio splendidus*, whose various ecological preferences were strong enough to trigger speciation. The samples showed the beginning of the sympatric speciation process. They sequenced 100 genomes from the samples and used their STARRIniGHTS software to infer recombination breakpoints. Alm discovered that most of the support for the zooplankton/free-living split is on an 18 kb operon responsible for zooplankton chitin catabolism.

## Experiences

Based on the organizer and attendee experiences at ISMB/ECCB 2009 as well as at earlier conferences, we have created a set of guidelines that can be used in the creation of live blogging policies for any life sciences conferences. These guidelines are available in a Perspective article published in concert with this report [2]. Both local and remote attendees found the live blogging helpful, informative, and a useful archive, and these guidelines should help support and direct future live blogging experiences.

Additionally, initial ISCB concerns over having an open live blogging policy were allayed after the success at this year's conference. Organizers initially feared that live blogging might distract from the conference. However, while the distraction of an Internet connection is one that exists whether or not live blogging is allowed, live blogging caused no discernible upset in the audience. Further, live blogging, while a useful tool for participating in topics of interest irrespective of physical presence, is no substitute for being at a conference where one can network, question the speakers face-to-face, and enjoy the stimulating buzz of activity. Indeed, the attendance at ISMB/ECCB 2009 was very close to the levels expected by the organizers. Organizers may also have been concerned that live blogging might produce uninformative content. However, this year's coverage at the ISMB/ECCB was very sober and largely efficient. Misuse of officially sanctioned microblogging areas, such as the addition of spam or rude comments, was another unfounded concern at ISMB/ECCB 2009, where all comments were pertinent, respectful, and polite.

## Summary

In last year's report on microblogging ISMB 2008 [1], the authors anticipated that new methods of using the Web and of reporting the conference would make live blogging even easier (http://www.bork.embl.de/~jensen/ismb2008/keynotes.php.html). This year, the ISMB/ECCB 2009 Web site contained all of the features of the mock-up, far more live bloggers participated than last year, and there was increased coverage of talks and special sessions. We, in turn, look forward to the new technologies appearing on the horizon (such as Google Wave), and how both tools and bloggers will make next year's conference an even greater success.

In summary, conference organizers found that microblogging added value for all conference attendees, and allowed attendees to follow the thoughts of others as well as to follow presentations that conflicted with others they wished to see. The usefulness of live blogging extends beyond the duration of the conference, remaining accessible long after the conference has closed.
